# N-Glycan profile analysis of transferrin using a microfluidic compact disc and MALDI-MS

**DOI:** 10.1007/s00216-016-9570-4

**Published:** 2016-04-30

**Authors:** Alessandro Quaranta, Anna Sroka-Bartnicka, Erik Tengstrand, Gunnar Thorsén

**Affiliations:** Department of Environmental Sciences and Analytical Chemistry, Stockholm University, Svante Ahrrenius v 12, 10691 Stockholm, Sweden; Department of Genetics and Microbiology, Maria Curie-Sklodowska Univeristy, Lublin, Poland

**Keywords:** Glycan pattern, Glycomics, N-linked oligosaccharide, Transferrin, Microfluidic, MALDI-MS

## Abstract

It has been known for a long time that diseases can be associated with changes to the glycosylation of specific proteins. This has been shown for cancer, immunological disorders, and neurodegenerative diseases. The possibility of using the glycosylation patterns of proteins as biomarkers for disease would be a great asset for clinical research or diagnosis. There is at present a lack of rapid, automated, and cost-efficient analytical techniques for the determination of the glycosylation of specific serum proteins. We have developed a method for determining the glycosylation pattern of proteins based on the affinity capture of a specific serum protein, the enzymatic release of the N-linked glycans, and the analysis of the glycan pattern using MALDI-MS. All sample preparation is performed in a disposable centrifugal microfluidic disc. The sample preparation is miniaturized, requiring only 1 μL of sample per determination, and automated with the possibility of processing 54 samples in parallel in 3.5 h. We have developed a method for the glycosylation pattern analysis of transferrin. The method has been tested on serum samples from chronic alcohol abusers and a control group. Also, a SIMCA model was created and evaluated to discriminate between the two groups.

## Introduction

Much research on the qualitative and quantitative measurement of glycans attached to proteins has been performed over the past decade [1]. This has occurred primarily because glycosylation patterns in a large number of glycosylated pharmaceutical biomolecules need to be determined and monitored. The glycosylation pattern of a specific therapeutic biomolecule may be important for both the efficacy and the safety of the pharmaceutical, as it can influence the interaction between the pharmaceutical and other biomolecules. This interaction, in turn, can affect the intended mode of action and the serum lifetime of the pharmaceutical or give rise to unwanted immunogenic properties [2].

Several strategies for protein glycosylation analysis have been presented in the literature [3]. Examples of such strategies include analysis of the intact glycoprotein using high-resolution MS [4], analysis of the glycopeptides using MALDI-MS [5] or LC-MS [6], and analysis of enzymatically released glycans using LC-MS or LC with fluorescence detection after derivatization [7, 8]. Moreover, other separation techniques, such as capillary electrophoresis [9] or anion exchange chromatography [10], have also been used in combination with pulsed amperometric, MS- or fluorescence detection. Some of these methods could be automated with a high degree of parallel processing to increase the sample throughput, but many require a separation step, using LC, for instance. This separation step leads to long processing times if many samples need to be analyzed, even under optimized conditions. Significant improvements in the time required for enzymatic release of glycans from proteins, as well as for liquid phase separation of fluorescently labeled glycans, have reduced the overall time required for the analysis [11]. Yet many protocols still use overnight digestion, time-consuming evaporation steps, and labeling reactions that require hours for completion.

The ability to process many samples in a short time is essential for validating the glycosylation patterns of specific serum proteins as biomarkers for a specific disease [11]. Several methods for high-throughput sample processing of glycoproteins for glycan analysis have been presented, but most of these do not address the entire process including the selective capture of the protein of interest. In most cases, the applications focus on glycan analysis of a wide range of proteins or analysis of biopharmaceuticals. One example of a highly parallel methodology that incorporates all necessary steps for sample pretreatment and analysis of the glycosylation of specific proteins is the microplate platform combined with a multichannel cGE, which has been used for the analysis of alpha-1-antitrypsin and protein A in a large number of samples [12]. Recent developments using bead-based sample preparation approaches in combination with multiplexed cGE analysis are promising for the glycan analysis of many samples processed in parallel [13].

Many consecutive sample preparation steps are used when analyzing N-glycans from specific proteins in biological samples [14, 15]. The following processes are usually required: separation of the desired proteins from other proteins in the sample, enzymatic digestion of the peptide backbone of the protein or enzymatic release of the attached glycans, glycopeptide enrichment or derivatization of the released glycans, separation of the glycopeptides or glycans, and detection using MS or fluorescence. Various cleanup steps are also necessary depending on the strategy used.

Gel electrophoresis techniques are unsuitable when isolating specific proteins from larger numbers of samples. Also, the risk of error increases when many manual steps are involved. This makes the use of integrated microfluidic devices or robotic handling platforms attractive for this type of analysis. The scientific literature presents several microfluidic devices: however, most address only some of the steps necessary for achieving glycan analysis from a crude sample [16].

An increased interest has lately been shown for the role of glycosylation in different diseases, and it has become more and more evident that protein glycosylation may be altered due to the presence of disease or during disease progression. Several types of cancer induce changes to protein glycosylation profiles [17]. Other diseases associated with protein glycosylation include autoimmune diseases [18, 19] and Alzheimer’s disease [20]. In the rare family of diseases known as congenital disorders of glycosylation (CDG), the altered glycosylation in itself is the cause of the disease, leading to symptoms such as psychomotor retardation [20].

Evidence of this association between certain diseases and protein glycosylation creates an opportunity for identifying new ways to diagnose or study disease progression. Haptoglobin, for instance, is a non-specific biomarker for liver diseases. The glycosylation state of haptoglobin, however, has proven a promising biomarker for hepatocellular cancer with good clinical sensitivity and specificity, especially if combined with the glycosylation state of alpha-fetoprotein [21]. One study suggests that monitoring the glycosylation state of prostate-specific antigens can reduce the number of false-positive results [22].

Transferrin is a common protein biomarker, useful for the diagnosis of bacterial infections. The protein has two sites where N-linked glycosylation occurs. The glycosylation state of transferrin has been used to diagnose chronic alcoholism and CDG. A change in the ratio between two different glycoforms of transferrin, “serum type” and “brain type” glycosylation, is believed to correlate to Alzheimer’s disease for certain population groups [20].

Previously, we proposed the use of a centrifugal-based microfluidic platform with the dimensions of a compact disc (CD) for glycan pattern analysis of therapeutic antibodies [23, 24]. All the sample preparation steps (from selective capture of the protein of interest from cell culture supernatant or serum to generation of crystals that are ready for analysis with MALDI-MS) can be automatically performed in the microfluidic CDs. The microstructures in the CDs have two miniature columns that can be packed with different sorbent phases, for example, affinity extraction and carbon phases. Particles with immobilized streptavidin are used to facilitate easy changes in the specificity of the affinity reagents used to capture the protein of interest.

The present study demonstrates the use of the microfluidic CD platform for selective capture of transferrin and subsequent enzymatic release (from the protein) and sample preparation of its N-linked glycans for MALDI-MS analysis. A biotinylated affibody [25] was used for the selective extraction of transferrin from serum samples. The newly developed method was applied to the analysis of the desialylated transferrin glycan pattern of a sample cohort of chronic alcoholics and control serum samples. The method is comparatively fast and, as desialylated glycans were studied, could complement the commonly used high-performance liquid chromatography (HPLC) method with fluorescence detection or other methods based on LC-MS analysis of glycopeptides. The architecture of the microfluidic platform is open in that the specificity of its microstructures can be easily changed by the use of different biotinylated affinity reagents. The platform architecture along with low consumption of samples and reagents provides a useful tool for analyzing glycosylation patterns of specific serum proteins.

## Materials and methods

### Materials

The EZ-link® Maleimide-PEG_2_-Biotin reagent, the Zeba™ desalting spin columns with a 7-kDa molecular weight cutoff, the porous graphitic carbon (PGC) HPLC column (purchased to obtain PGC particles of the right dimension), and the Normal Mouse Serum used as blank were obtained from Thermo Scientific (Waltham, MA, USA). The affibody with affinity for human transferrin was purchased from Affibody AB (Solna, Sweden). The following reagents were obtained from Sigma-Aldrich (St. Louis, MO, USA): 2,5-dihydroxybenzoic acid (DHB), dimethylaniline (DMA), dithiothreithol (DTT), ethylenediaminetetraacetic acid (EDTA), Tween 20, standard human transferrin, and the commercially available N-linked glycan standards H5N4 ([(Gal-GlcNac)_2_Man_3_(GlcNac)_2_]) and Man3 ([2Man_3_(GlcNac)_2_]).

The Peptide N-Glycosidase F (PNGase F) kit (for enzymatic release of N-linked glycans) and the α-2-3,6,8 Neuraminidase kit for release of sialic acids were obtained from New England Biolabs UK Ltd. (Hitchin, UK). Sodium dihydrogen phosphate, disodium hydrogen phosphate, and sodium chloride were obtained from Merck (Darmstadt, Germany). Acetonitrile (ACN), methanol, and isopropanol were purchased from Rathburn Chemicals Ltd. (Walkerburn, UK). All solvents were of analytical grade unless otherwise stated, and water was purified using a Millipore water purification system to a resistance >18 MΩ/cm. A prototype Gyrolab instrument (model PP2.1) equipped with the same hardware as the commercially available instruments, streptavidin-coupled particles, and microfluidic CDs were provided by Gyros AB (Uppsala, Sweden).

### Human serum samples

Human serum samples from control (*n* = 12) and chronic alcoholic (*n* = 19) subjects were provided by Akademiska Sjukhuset (Uppsala, Sweden), with ethic committee approval from Ups 01-367. Once received, the samples were diluted 9:10 with water containing 0.1 % Tween 20, aliquoted, and stored at −20 °C until further use. Sample dilution with Tween 20 proved necessary for lowering the serum’s surface tension, thus improving its behavior inside the microfluidic system.

### Biotinylation of the affinity reagent

The anti-transferrin affibody was biotinylated according to a biotinylation protocol provided by Gyros AB. The following procedure was used: 500 μg of lyophilized affibody was dissolved in 500 μL of reduction buffer consisting of 50 mM sodium dihydrogen phosphate, 50 mM disodium hydrogen phosphate, 2 mM EDTA, and 150 mM sodium chloride at pH 7.5. DTT was added to a final concentration of 0.5 M. The reduction was performed under agitation for 2 h at room temperature, and the excess of reducing agent was removed using 7-kDa cutoff Zeba™ spin columns. The spin columns were conditioned with the appropriate derivatization buffer prior to the loading of the affibody. The reduced affibody was mixed with a solution containing a 12-fold molar excess of Maleimide-PEG_2_-Biotin reagent, and the labeling reaction was allowed to proceed overnight on ice. The excess of biotinylating reagent was removed by centrifugation using 7-kDa cutoff Zeba™ spin columns. Performing the reduction at room temperature prevented the affibody from denaturing, allowing the DTT to reduce only the sulfhydryl group that connects the two identical subunits that the anti-transferrin affibody consists of (personal communication, Gyros AB and Affibody).

### Microfluidic platform

The design and basic operation of the microfluidic CDs have been described in a previous publication [24]. The microfluidic CDs used in this study contain 54 structures divided into 6 segments of 9 structures each. Each structure has a sample inlet reservoir that can hold up to 1 μL of sample. This reservoir is connected to an affinity column packed with 15-μm streptavidin-coupled particles on which biotinylated affinity reagents can be immobilized. A liquid router downstream of the streptavidin columns can direct the flow into a waste channel or into a second column packed with a carbon stationary phase, depending on the rotational frequency used to move the liquid. Also, an inlet for reagent solutions that has a volume definition unit is connected to the second column. This unit can be used to precisely meter 200 nL of reagent solutions. At the exit of the second column, there is an evaporation unit where crystals amenable for MALDI-MS are formed in a confined area.

Briefly, the following operations were performed on the microfluidic CDs: capture of the biotinylated anti-human transferrin affibody on the streptavidin-coated beads; selective capture of transferrin from human samples; simultaneous release of the N-linked glycans from the transferrin, and of the sialic acids from the glycans, using a mixture of PNGase F and neuraminidase; recapture of the released desialylated glycans on the carbon columns and selective elution of the glycans; and co-crystallization with the MALDI matrix.

The spin sequences used to control movement of the liquids in the microfluidic structures were the same as described in the previous paper [23], except for the rotational frequencies used for elution of the glycans from the carbon columns. These frequencies were slightly altered due to a change in the material used in the carbon columns from a poly-disperse 400-mesh graphitic carbon black (GCB) to a mono-disperse 7-μm-diameter PGC material obtained from an HPLC column.

### Release of glycans in the CD platform

**Fig. 1:**
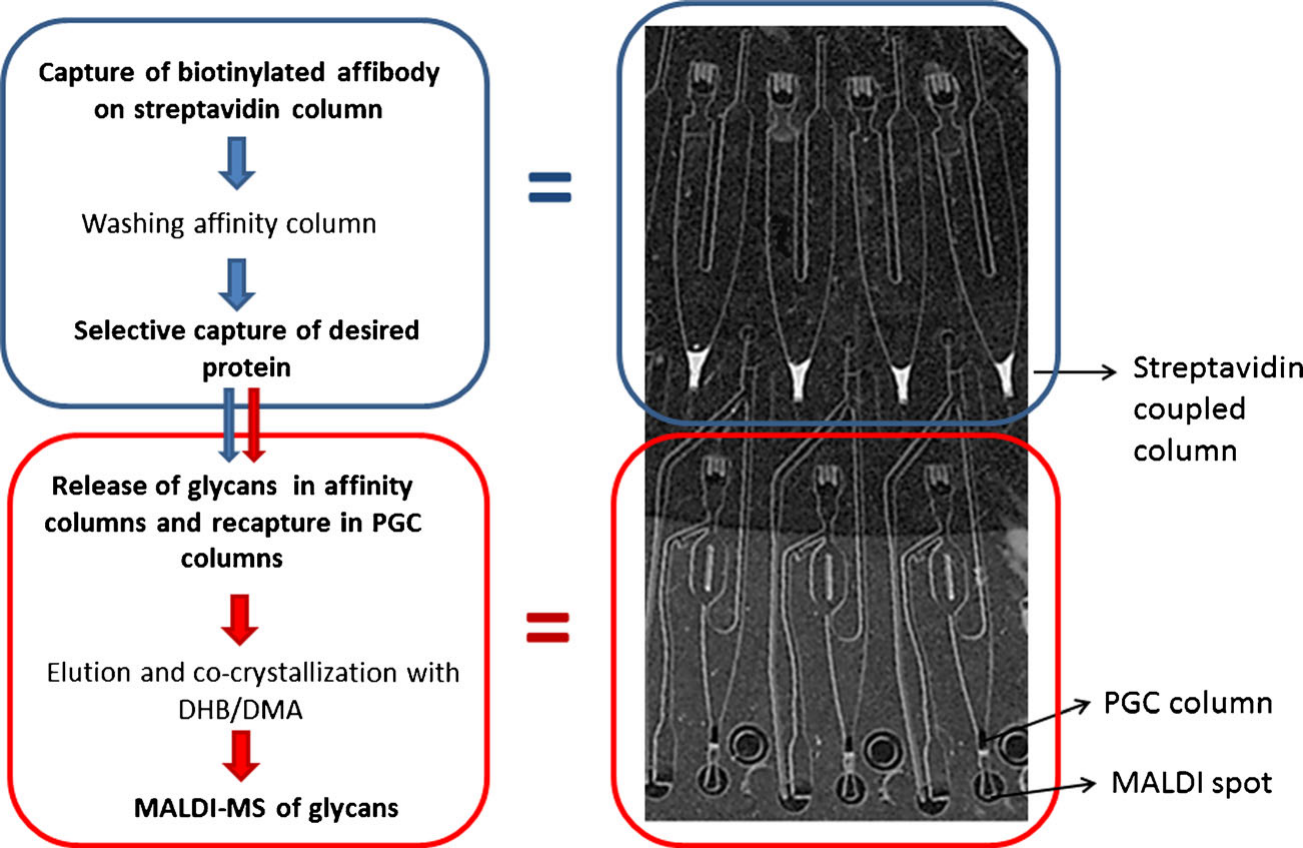
Workflow of the method and a picture of the microfluidic structures, with the streptavidin and carbon columns shown

The inner columns were packed with streptavidin-coupled particles, and the outer columns were packed with PGC particles after first washing all the structures with water and with 50 % isopropanol in water. Once the CD was ready, the samples were treated according to the workflow displayed in Fig. 1.

The streptavidin columns were first conditioned with 0.7 μL of phosphate-buffered saline solution (PBS, pH 6.8) before 1 μL of a 42-μg/mL solution of biotinylated anti-transferrin affibody was loaded into each structure. Affibody binding to the streptavidin was performed by spinning the CD at 700 rpm for 120 s and at 2200 rpm for 360 s. Low rotational frequencies were used in this step to direct the liquids to the waste channel. After washing the inner column with PBS, 1 μL of human serum sample was loaded into each structure and transferrin was selectively captured using the same spinning program as that used for affibody binding. All other proteins and plasma components were sent to the waste channel. After transferrin capture, the inner columns were washed thrice with 1 μL of PBS.

Before loading the mixture of enzymes for glycan release and desialylation, the carbon columns were conditioned four times with 0.6 μL of 50 % ACN aqueous solution and twice with 0.6 μL of deionized water.

Enzymatic cleavage of the glycans from the protein was performed by loading 0.35 μL of the mixed enzyme solution (consisting of PNGase F and neuraminidase, 5:1 U) into each structure. A small portion of this solution was moved into the first column using a short spin step at 3600 rpm, and was kept in position by lowering the spinning frequency to 400 rpm for 15 min, allowing the enzymatic reaction to proceed. After this step, the frequency was suddenly increased to 3600 rpm again to move another portion of the enzyme solution forward, and to direct the released glycans to the carbon column, where they were retained. This process was repeated four times, giving a total reaction time of 1 h.

Released and desialylated glycans were then desalted using two additions of water, after which the captured glycans were finally eluted and co-crystallized with a solution of 50 % ACN in water containing the MALDI matrix (21.5 mg/mL DHB and 4.6 mg/mL DMA). In each structure, 0.45 μL of this solution was loaded, and a 0.20-μL volume was defined in the volume definition unit and moved through the carbon column. A number of strong rotational pulses (four, at 1600 rpm) with intermittent low rotational frequency were used to ensure that all the elution liquids had established a meniscus at the opening of the channel into the evaporation structure. The last pulse was also used to push out a drop of eluting solution which was then held in place using a rotational frequency of 800 rpm for 5 min. At this speed, the centrifugal force pushing the drop outside the CD and the capillary action pulling the liquid back in the structure were balanced, and the drop was held in position while the solvent evaporated. This process led to the formation of crystals containing the analytes and the matrix, ready for the MALDI-ToF analysis.

### MALDI-ToF-MS analysis for glycan profiling

Pieces suitable for mounting on the dedicated sample holder were cut from the CD using a dedicated punching tool. The pieces were inserted into the sample holder two at a time. Mass spectra were acquired using a Voyager-DE™ STR MS instrument (Perspective Biosystem, Framingham, MA, USA), equipped with a nitrogen laser (337 nm) and operated in reflector mode with positive ionization. Before each analysis, the instrument was calibrated using a mixture of the Man3 and H5N4 glycan standards, dispensed in reference positions close to each evaporation well. Acquisition was performed with an accelerating potential of 20 kV, a grid voltage set to 60 %, and a delay time of 150 ns. The laser intensity was set to the lowest value that produced strong glycan peaks, keeping the noise level low. Spectra were acquired in a mass range from *m*/*z* 800 to *m*/*z* 3000, with a low mass gate set at *m*/*z* 700. All spectra, resolutions, and *S*/*N* ratios were processed and calculated by the Data Explorer V4 software (Applied Biosystems Inc., Foster City, CA, USA).

### MALDI-MS imaging

All the MALDI mass spectrometry imaging (MSI) experiments were performed using a Bruker Ultraflextreme II MALDI-TOF/TOF-MS instrument (Bruker Daltonics, Bremen, Germany) equipped with a Smartbeam II 2-kHz laser. MSI data were visualized using FlexImaging (Bruker Daltonics, Bremen, Germany) version 4.1 for TOF/TOF data. Positive ion mode was used, and the spectra were acquired in the mass range 700–3500 *m*/*z*. Regions of interest were manually selected using both optical and MS images. The spatial resolution was set to 50 μm. Parts of the CD were inserted into a holder with the same dimensions of a standard Bruker MALDI target (provided by Gyros).

### Automatic data integration

The automatic integration procedure used for extracting data from each spectrum makes use of five data points, in close proximity of the intended mass of the different glycans. This method was used as it had previously been experimentally determined to be the most robust in comparison with other integration algorithms. The other methods were based on the numerical integration of different regions in the proximity of the expected masses of the glycans, for instance integrating 50 data points, corresponding to 1.2 Th (*m*/*z*), towards the low mass region and 50 data points towards the high-mass region. This was used in previous work in order to capture similar isotopic patterns for signals close to the detection limit and signals with high intensity [26].

## Results and discussion

Previously described methods for glycan pattern analysis with the microfluidic discs have either used immobilized protein A or streptavidin particles for the affinity capture in the first column. Use of protein A particles to capture therapeutic immunoglobulin G (IgG) molecules facilitates deglycosylation directly in the affinity column, as protein A is not a glycoprotein and will not in itself contribute with interfering glycans. The affibody used in the present study was also not glycosylated; thus, the same simple strategy as these previously described methods could be used. If a glycosylated affinity reagent is used, the alternative is to elute the protein prior to enzymatic processing [27] or remove the glycans from the capturing reagent before using it [23].

An overnight incubation at 37 °C is typical practice to ensure a quantitative deglycosylation. However, other studies show that 60 min is an adequate time for a complete deglycosylation of the target immobilized protein [28, 29]. Even shorter deglycosylation times have been presented in the literature [11]. Performing the deglycosylation in 60 min allowed us to treat 54 samples in parallel for a total duration of 3.5 h for each microfluidic CD.

### MALDI-MS results

**Fig. 2:**
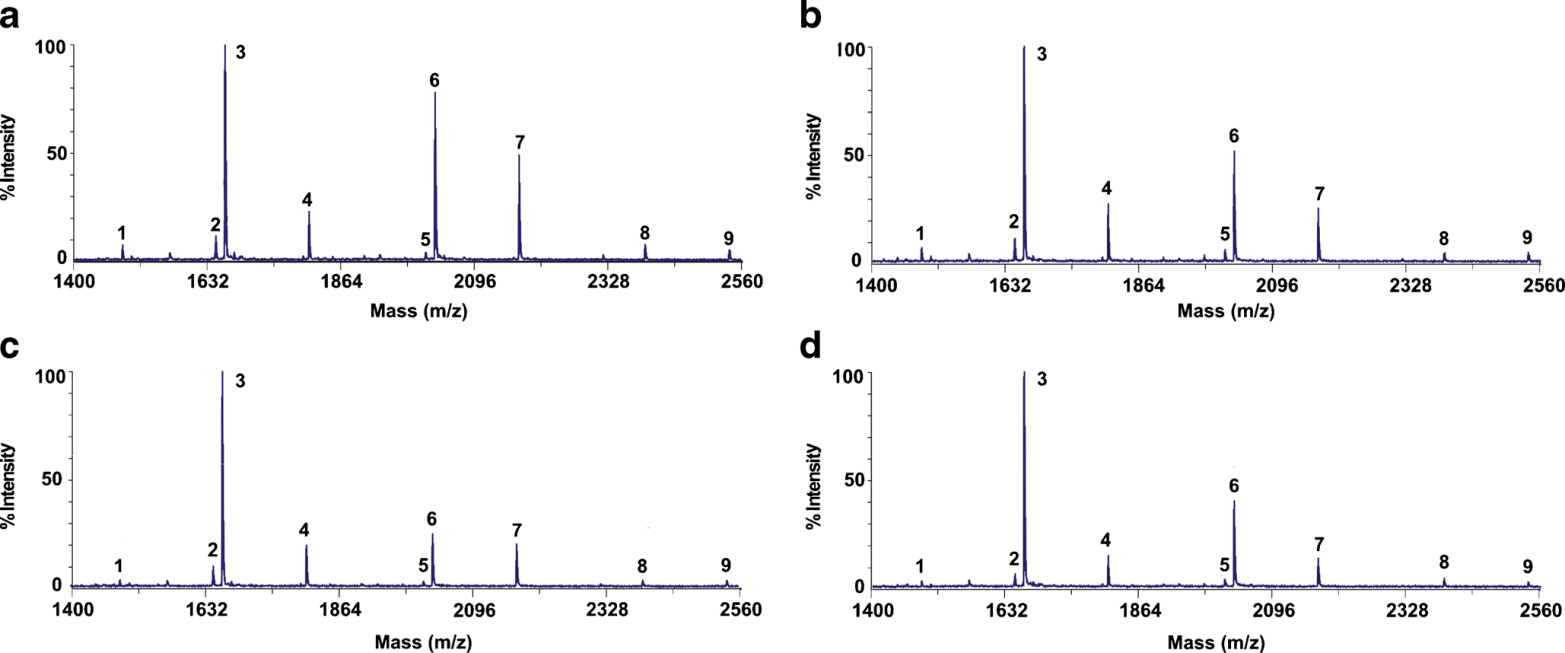
MALDI-ToF spectra of desialylated transferrin glycans from **a** male alcoholic, **b** male control, **c** female alcoholic, and **d** female control

Representative glycan patterns of transferrin from the serum of control and chronic alcoholic subjects are shown in Fig. 2. The glycan structures identified are reported in Table 1. The reported structures were identified based on the mass of the glycans and on prior reports in the literature [21, 30]. All glycans were detected as sodium adducts.

**Table 1:**
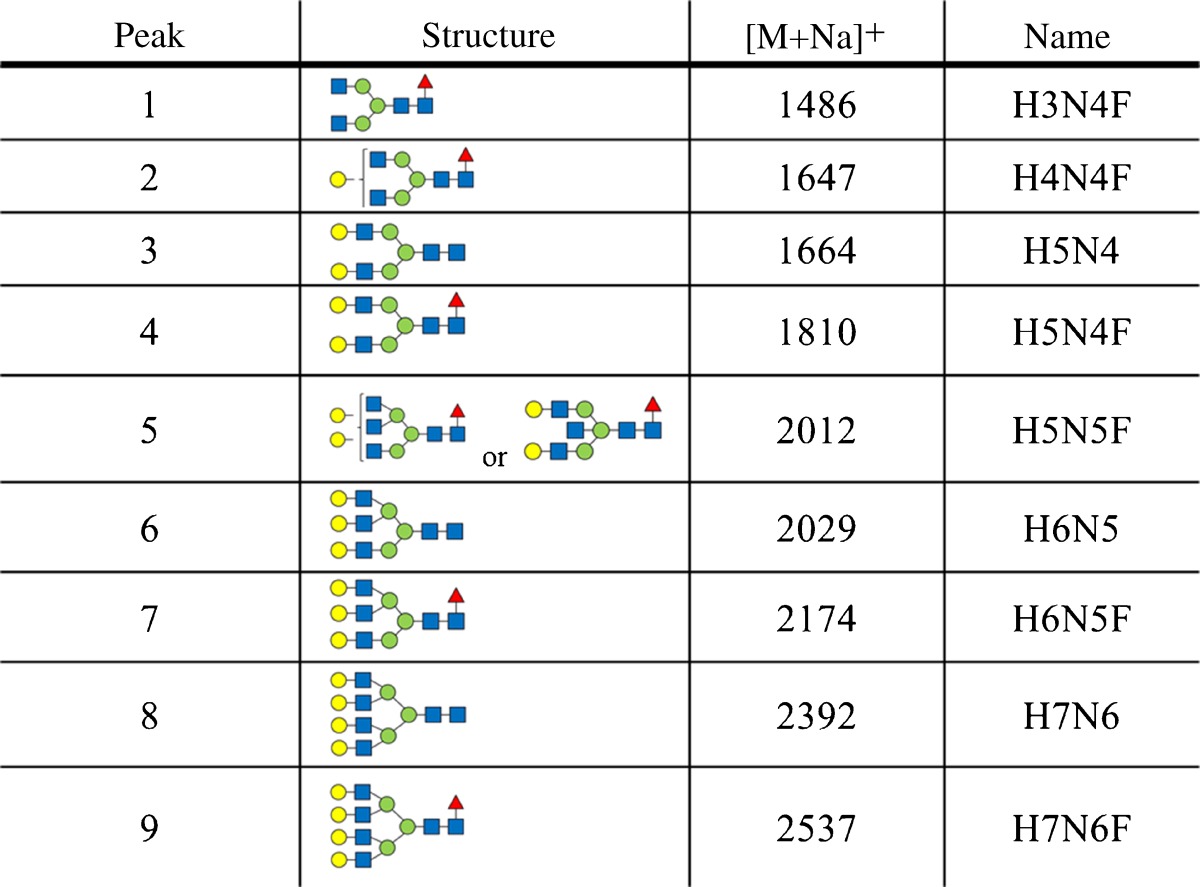
Structures, *m*/*z* values, and names for the observed glycans (red triangle, fucose; blue square, *N*-acetyl glucosamine; green circle, mannose; yellow circle, galactose)

The non-fucosylated biantennary glycan (*m*/*z* 1664, peak 3) was the most abundant glycan of all the samples analyzed. A total of nine glycan structures were observed in all the samples, corresponding to di-, tri-, and tetra-antennary glycans that were both mono- or non-fucosylated. The same glycans, with the exception of peaks 1, 2, 5, and 9, were also present on the standard transferrin that we analyzed.

### Selectivity

To evaluate the selectivity of the developed method, a blank consisting of standard mouse serum was analyzed. This blank was a suitable analyte-free matrix since the anti-transferrin affibody does not cross-react with murine transferrin. Any glycans detected would have originated from non-specifically bound proteins. No signals corresponding to glycans could be detected in any of the replicate analyses of this serum (*n* = 6). This observation demonstrates the satisfactory selectivity of the method. Standard human transferrin was spiked into the mouse serum at relevant serum concentrations and captured by the affibody as a positive control. Strong signals for all the glycans present on the standard transferrin were detected in all replicates of this sample.

Spectra from human subjects, shown in Fig. 2, show traces of glycans present on, for instance, IgG. These proteins contain mostly fucosylated biantennary glycans, especially H3N4F, H4N4F, H5N4 (which was the most intense), and H5N4F, as well as some sialylated variants of these [31]. Synthetic protein mixtures, such as IgGs and haptoglobin with HSA, were analyzed at relevant serum concentrations as additional controls. These samples showed minor traces of glycan signals arising from non-specifically bound proteins, but the intensities of the signals were never more than 10 % of the positive transferrin controls.

The complete absence of glycans coming from IgGs in the mouse serum is probably related to the lower content of these proteins in this matrix. Reference mouse serum usually contains between 30 and 70 % less IgGs than normal human serum. Mouse serum is therefore not an ideal blank. However, it is a more representative blank compared to a mixture of standard proteins, as it contains all the components expected in a serum sample. Transferrin-depleted human serum would constitute the best possible blank for the experiment but is difficult to achieve without altering the composition of the serum in other ways, for example through dilution.

**Fig. 3:**
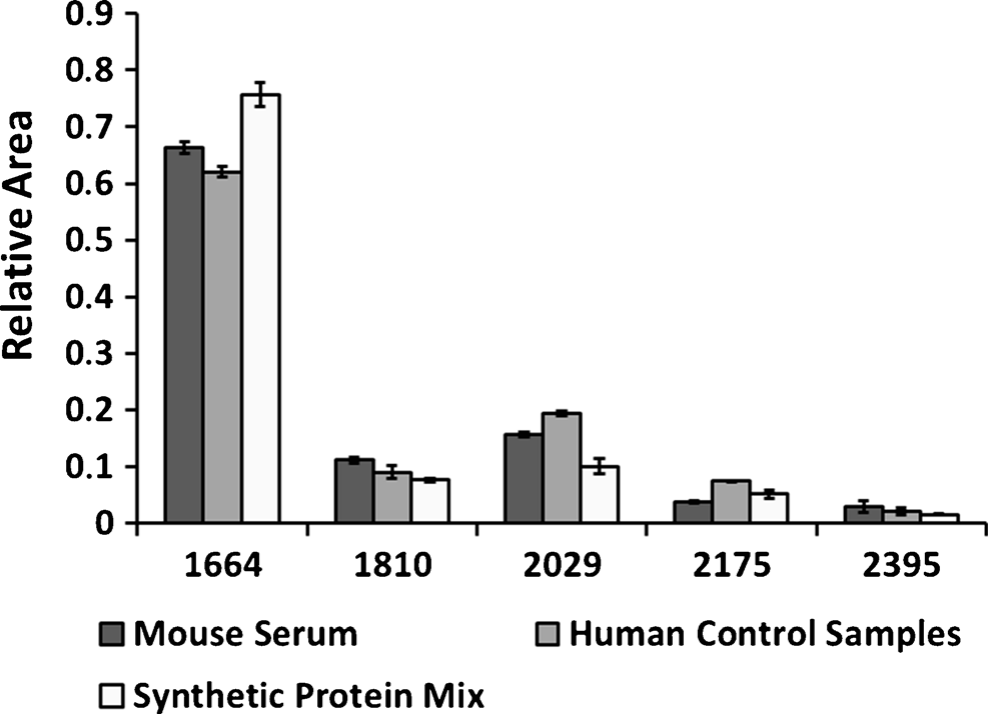
Relative areas for the five identified glycans of the standard transferrin spiked into mouse serum and synthetic protein mix, and comparison with the control serum sample

The glycosylation pattern determined for human serum samples from the control group was in good agreement with the glycosylation pattern of the standard transferrin spiked into mouse serum. When the standard transferrin was spiked into the synthetic protein mixture, the glycosylation pattern shows a slight alteration, with higher relative abundance of the non-fucosylated bisecting glycan and a decreased abundance of the non-fucosylated triantennary. In Fig. 3, a comparison of the relative areas for the five glycans found in standard transferrin, after spiking it into mouse serum and the synthetic protein mix, is shown. These patterns are compared to the analysis of a human control sample.

### Use of mixed enzyme

Previous studies performed using the microfluidic CD system analyzed the glycan pattern of recombinant therapeutic antibodies. The glycans present on these proteins did not have terminal sialic acids to any significant extent, so the analysis could be performed in positive ionization mode. Glycans on human transferrin have terminal sialic acids. Although the sialic acid content has been reported as a potential biomarker in breast, ovarian, and liver cancer [17], the presence of these residues on the antennae of the cleaved glycans can add heterogeneity to the obtained spectra. Heterogeneity occurs because glycans with the same core structure will give rise to signals at different *m*/*z* values due to their different level of sialylation. Removing the sialic acids before the analysis will remove this complexity and improve the general sensitivity as the glycans with the same core but different sialic acid content will merge into a single peak [32].

Information concerning the sialylation level of transferrin will be lost using the abovementioned approach, but this methodology is suitable if the information sought pertains to the core structure or branches of the glycan. This is the case for many of the changes to N-linked glycans attached to proteins that have been presented so far [17]. In the present method, neuraminidase has been added together with PNGase F to the samples, producing high-intensity signals and easily construable spectra. Different ratios of enzymes and enzyme concentrations have been tested to find the most suitable enzyme mixture.

### Short-term and intermediate precision

A nested ANOVA with random effects was used to identify the most significant source of imprecision and to calculate any contributions to imprecision arising from the same sample being analyzed on different CDs, replicates within one CD, and replicate MALDI-MS analysis within each sample spot. The software used for the calculations was R. The data set contained two samples, three CDs, three replicates of each sample per CD, and three summed spectra from different locations within the sample spot. The relative areas of the four most intense glycan signals were used for the calculations, as some of the weaker signals were lacking in one or more of the replicate analyses. There was no significant contribution to imprecision from the samples, e.g., the glycan patterns of the two samples could not be differentiated, and the relative standard deviations (RSDs) presented in the next paragraph were calculated on the basis of the average relative area from these two samples.

Significant contributions to imprecision came from the sample replicates within the CDs, with RSD values of 7.0, 8.3, 10.5, and 10.0 % for the H5N4, H5N4F, H6N5, and H6N5F glycans respectively. Contributions to imprecision arising from the replicate analysis within each sample spot were 3.4, 9.8, 5.1, and 7.3 % RSD. The between-CD contribution was not significant, as it was overshadowed by the within-CD and within-sample spot contributions. Assuming the different contributions, within-CD and within-sample spot, to be independent effects facilitates the calculation of the overall imprecision for the different glycan signals as the square root of the sum of the variance contributions of these effects. This gave overall imprecision values of 7.8, 12.8, 11.7, and 12.4 % RSD respectively. It may have been more suitable to calculate the standard error, since all the data used in the calculations were based on replicate analysis. Standard error calculations would, of course, have reduced the imprecision, but it is more common to present the RSD values, as we have done here.

There are several possible reasons for the variation in relative abundances between replicates. Differences in the manually packed carbon columns may influence the elution of the glycans from the PGC columns so that the structures have slightly different elution profiles, i.e., the area of the evaporation structure that is covered by matrix and analyte crystals. These different elution profiles could, in turn, affect the crystallization process and give rise to some variation. Improvements in the packing processes used and improved quality control of the prototype discs will probably alleviate these problems.

Beyond the CD-related issues, another contribution to variance can come from the mass spectrometer. The MALDI-ToF instrument used in this work had a short linear response, about two orders of magnitude. A short linear response may result in incorrect relative areas if the most intense peak is outside the linear range or if some peaks are too low to be measured. In the present study, spectra with signals that were too intense or too low were discarded and reanalyzed.

### MALDI imaging

The MALDI-MSI was used to assess the distribution of the matrix and analyte in the microfluidic sample spots. The imaging techniques can directly visualize the location of target compounds and determine their relative abundance and spatial distribution [33]. The homogeneity of the sample spot, and thus the repeatability of the obtained spectra inside each spot, is a very important factor for successful implementation of an automated workflow using the microfluidic CDs [34].

Co-crystallization of the target analyte and polymorphism of the MALDI matrix can be studied using several techniques: e.g., solid-state NMR [35, 36], Raman imaging [37, 38], confocal fluorescence microscopic imaging [39], and MS imaging [40, 41]. Moreover, a variety of other techniques can determine the homogeneity of the crystals and the effectiveness of the desorption and ionization processes [42]. Non-homogeneous co-crystallization of glycopeptides within the MALDI sample standard spot has previously been reported using the MS imaging technique [40, 41]. In this study, MALDI-MSI was used for studying the homogeneity of the released glycans in the microfluidic CD sample spots.

**Fig. 4:**
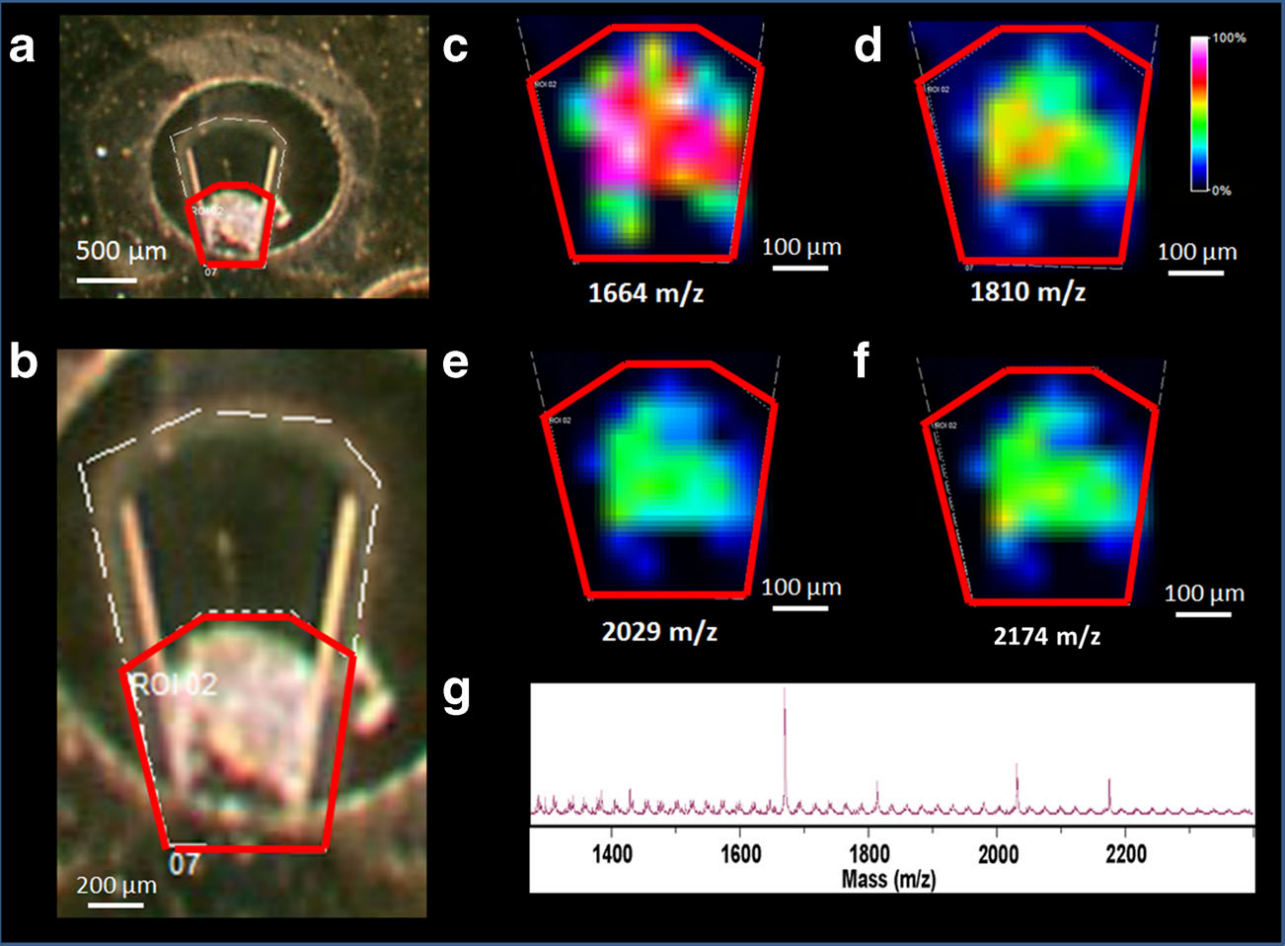
MALDI-MSI images showing distributions of the maximum intensity in the range of specific glycans in the microfluidic device sample spot, from serum alcoholic samples: **a** optical image of the CD microfluidic sample spot, **b** magnification of the sample spot with the *marked area* of mapping the ROI (region of interest), **c** H5N4 at 1664 *m*/*z*, **d** H5N4F at 1810 *m*/*z*, **d** H6N5 at 2029 *m*/*z*, **f** H6N5F at 2174 *m*/*z*, **g** averaged spectrum from the *image area* marked by the ROI

Figure 4a, b shows the optical image of the whole sample with the region of interest (ROI) that was examined for this structure encircled in red. The whole ROI was scanned and the signal distributions of the four most intense glycans of transferrin: H5N4 at 1664 *m*/*z*, H5N4F at 1810 *m*/*z*, H6N5 at 2029 *m*/*z*, and H6N5F at 2174 *m*/*z* are presented in Fig. 4c–f. The most intense signal in this sample was 1664 *m*/*z*, which correlates with the color code distribution image in Fig. 4c. The other glycan signals at 1810 *m*/*z*, 2029 *m*/*z*, and 2174 *m*/*z* had weaker intensities, but the signal distribution within the ROI was the same for each image. This is important, as the spectra will have the same relative intensity as the glycan signals, but with different absolute intensity, independent of where in the sample spot the MALDI spectrum is collected. Figure 4b also shows a dense and homogeneous coating of small crystals. Glycan signals are present over the entire area covered by crystals, demonstrating a fairly homogeneous co-crystallization that is beneficial for an automated workflow. Figure 4g shows the summed mass spectra over the entire ROI. Eight different sample spots were investigated using MALDI-MSI, each giving similar results to the sample spot shown in Fig. 4. This result correlates well with the results from the ANOVA for the within-sample spot imprecision.

### Analysis of clinical samples using the developed method

The developed method was applied to the analysis of two sample sets: serum samples from chronic alcoholic patients and a control group. We aimed to test whether the method could be used to discriminate between these two groups based on their desialylated glycan patterns. Peak intensities were used in the data evaluation, and automated extraction of data from the spectra was based on five data points in the mass spectra in relative proximity to the expected *m*/*z* value of the glycan signal. Different integration- or peak intensity determination methods were compared prior to the evaluation of the data, and the method we used was determined to be the most robust method for automated data processing. Baseline correction was performed on each spectrum before exporting the data in a tabulated text format for further processing in MatLab.

The numbers of samples in each group are 12 and 19, respectively, for control and alcoholics. No statistical difference between the average values for the different glycan signals in the different groups could be observed, but we could observe that the variation in relative intensity for the different glycans was larger for the chronic alcoholics than for the controls. This was especially evident for the H5N4 (1664) and H6N5 (2029) signals, which had a statistically significant larger variation in relative peak areas. Similar average values were obtained for the alcoholic and the control groups (0.45 and 0.53 respectively) when a ratio between the relative areas of H5N4 and H6N5 was considered. Variability observed in control samples can be explained by personal variations in the glycosylation pattern and by the variation introduced by the sample processing and MALDI-MS analysis yielding a total RSD for the control group of 13 %. For the alcoholic samples, the values were spread much wider, resulting in a total RSD value of 51 %.

**Fig. 5:**
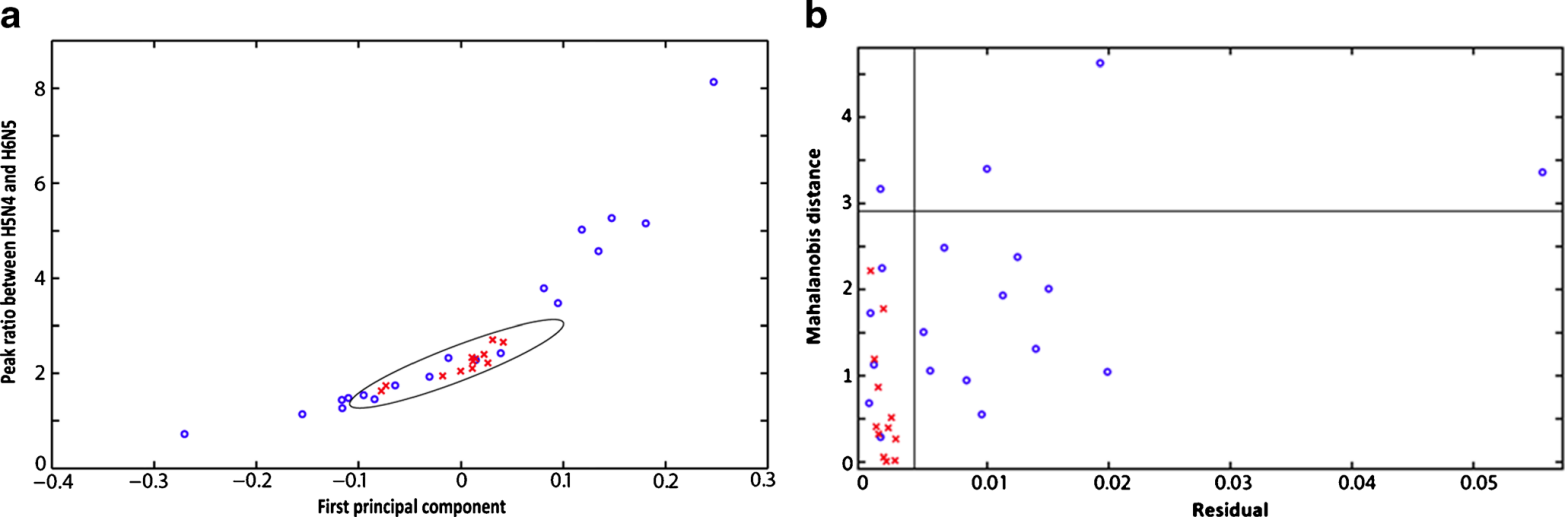
**a** PCA and **b** SIMCA plots of the analyzed samples. The control samples (*red x’s*) have been used to create a SIMCA model. Samples that had sufficiently low Mahalanobis distance to the center of the model, and sufficiently low residual, were classified as non-alcoholics. Here, 14 of the samples from the alcoholics (*blue circles*) fell outside the decision limit and were correctly classified

The generated dataset was analyzed using multivariate data analysis methods. The more varied glycosylation patterns of the alcoholics were used for classification of the groups, both univariate with the ratio between the H5N4 and H6N5 glycans and multivariate using principal component analysis (PCA) [43] and soft independent modelling of class analogy (SIMCA) [44]. Using a 95 % confidence interval for the control group as a decision limit, 12 of the 19 alcoholics were correctly classified using PCA (Fig. 5a). Since the alcoholics had a larger data variation, SIMCA was a more suitable classification method than PCA. A SIMCA model was constructed based on the control group, and samples from the alcoholic cohort were classified as belonging or not belonging to the control group. The results from the SIMCA analysis are shown in Fig. 5b. Of the alcoholic cohort, 14 of the 19 samples were correctly classified, indicating that the residual provides relevant information. A repeated fourfold segmented cross-validation of the SIMCA model gives 4 % false positives and 40 % false negatives.

The limitation of SIMCA is that it is a single class classifier: it only models one class. This limitation would normally be an issue in situations with two classes. Here, it was an advantage, as variation in the class of alcoholics was very widely spread: the variation in the alcoholics encompasses the variation of the control group. Other classification methods, such as LDA, can be troublesome for accurately assigning classes, unless the residual is accounted for. Given the results from the PCA, it is possible to use such a classifier, but then the model would require more samples from the alcoholics to fully cover the variance in that group.

Classical approaches to determine chronic alcoholism in people are based on quantification of the carbohydrate-deficient transferrin (CDT) or on measurement of the ratio between CDT and γ-CDT [45, 46]. Considering clinical sensitivity, calculated as the ratio between the number of true positives and the sum of true positives and false negatives, as the ability of a test to correctly identify patients with a disease, the values reported for CDT and γ-CDT are 79 and 75 % respectively [46, 47]. The method proposed here, still only a test of the developed microfluidic method on a very limited sample set, performed comparably to classical approaches in positively recognizing chronic alcoholism, producing a clinical sensitivity value of 74 %. The proposed method, however, has the advantages of very short durations, a high level of automation, and small amounts of reagents and samples required.

## Conclusions

We have presented a rapid, miniaturized method for glycan analysis of a selected serum protein, transferrin, based on affinity capture of the target protein and enzymatic release of the glycans with subsequent MALDI-MS analysis. All sample preparation steps are automated, and few manual steps are involved in the entire procedure. Automation reduces the manual processing workload and the probability of errors associated with human operators. The sample processing is fast, with a single CD being processed in 3.5 h, not including the MALDI-MS analysis. Use of mixed enzymes to simultaneously release the glycans and sialic acids yields easily interpreted spectra that give information concerning the relative abundances of core glycan structures on the protein of interest. The miniaturization greatly reduces the consumption of expensive affinity reagents and enzymes, as reported previously [23].

The analytical performance of the proposed method was investigated by evaluating the imprecision of the relative areas, which was less than 15 % RSD for the major glycan signals. MS imaging was used to investigate the spatial distribution of glycans within the CD sample spots, as well as to determine the homogeneity of the sample spots. The glycan pattern of standard human transferrin spiked into control mouse serum was very consistent with the glycan pattern of transferrin in human control samples.

The developed method was applied to the analysis of serum samples from chronic alcoholics and control subjects. While the control serum samples showed a relatively stable glycosylation pattern, with variations consistent with the normal interpersonal variability, alcoholic serum samples exhibited variability that exceeded the normal limits. These observations were used to create a model that could discriminate between samples from chronic alcohol abusers and control subjects, albeit with a high degree of false positives.

The selectivity of the affinity column can easily be changed by replacing the affinity binder, and different affinity binders may be used in different structures for measuring glycosylation patterns of several proteins within a single CD. We believe the results of this study show that the microfluidic CD method can be fast, compared to classical approaches, and can be useful for studying glycan biomarkers in serum samples.

## References

[1] Novotny MV, Alley WR (2013). Recent trends in analytical and structural glycobiology. Curr Opin Chem Biol.

[2] Beck A (2014). Biosimilar, biobetter and next generation therapeutic antibodies. MAbs.

[3] Pabst M, Altmann F (2011). Glycan analysis by modern instrumental methods. Proteomics.

[4] Rose RJ, Damoc E, Denisov E, Makarov A, Heck AJ (2012). High-sensitivity Orbitrap mass analysis of intact macromolecular assemblies. Nat Methods.

[5] Papac D, Wong A, Jones AJS (1996). Analysis of acidic oligosaccharides and glycopeptides by matrix-assisted laser desorption/ionization time-of-flight mass spectrometry. Anal Chem.

[6] Wuhrer M, Deelder AM, Hokke CH (2005). Protein glycosylation analysis by liquid chromatography-mass spectrometry. J Chromatogr B Anal Technol Biomed Life Sci.

[7] Guile G, Rudd PM, Wing DR, Prime SB, Dwek RA (1996). A rapid high-resolution high-performance liquid chromatographic method for separating glycan mixtures and analyzing oligosaccharide profiles. Anal Biochem.

[8] Mittermayr S, Bones J, Doherty M, Guttman A, Rudd PM (2011). Multiplexed analytical glycomics: rapid and confident IgG N-glycan structural elucidation. J Proteome Res.

[9] Callewaert N, Van Hecke A, Schollen E, Matthijs G, Contreras R (2001). Carbohydrate electrophoresis on the DNA-sequencer: technology development and first applications. Glycobiology.

[10] Hardy M, Townsend R (1988). Separation of positional isomers of oligosaccharides and glycopeptides by high-performance anion-exchange chromatography with pulsed amperometric detection. Proc Natl Acad Sci U S A.

[11] Shubhakar A, Reiding KR, Gardner RA, Spencer DI, Fernandes DL, Wuhrer M (2015). High-throughput analysis and automation for glycomics studies. Chromatographia.

[12] Ruhaak LR, Koeleman CA, Uh HW, Stam JC, van Heemst D, Maier AB (2013). Targeted biomarker discovery by high throughput glycosylation profiling of human plasma alpha1-antitrypsin and immunoglobulin A. PLoS One.

[13] Varadi C, Lew C, Guttman A (2014). Rapid magnetic bead based sample preparation for automated and high throughput N-glycan analysis of therapeutic antibodies. Anal Chem.

[14] Sturiale L, Barone R, Palmigiano A, Ndosimao CN, Briones P, Adamowicz M (2008). Multiplexed glycoproteomic analysis of glycosylation disorders by sequential yolk immunoglobulins immunoseparation and MALDI-TOF MS. Proteomics.

[15] Bones J, Mittermayr S, O’Donoghue N, Guttman A, Rudd PM (2010). Ultra performance liquid chromatographic profiling of serum N-glycans for fast and efficient identification of cancer associated alterations in glycosylation. Anal Chem.

[16] Lazar IM, Deng J, Ikenishi F, Lazar AC (2015). Exploring the glycoproteomics landscape with advanced MS technologies. Electrophoresis.

[17] An HJ, Kronewitter SR, de Leoz ML, Lebrilla CB (2009). Glycomics and disease markers. Curr Opin Chem Biol.

[18] Mackiewicz A, Mackievicz K (1995). Glycoforms of serum alpha-1-acid glycoprotein as markers of inflammation and cancer. Glycoconj J.

[19] McCarthy C, Saldova R, Wormald MR, Rudd PM, McElvaney NG, Reeves EP (2014). The role and importance of glycosylation of acute phase proteins with focus on alpha-1 antitrypsin in acute and chronic inflammatory conditions. J Proteome Res.

[20] Barone R, Sturiale L, Palmigiano A, Zappia M, Garozzo D (2012). Glycomics of pediatric and adulthood diseases of the central nervous system. J Proteome.

[21] Zhu J, Lin Z, Wu J, Yin H, Dai J, Feng Z (2014). Analysis of serum haptoglobin fucosylation in hepatocellular carcinoma and liver cirrhosis of different etiologies. J Proteome Res.

[22] Meany D, Zhang Z, Sokoll LJ, Zhang H, Chan DW (2008). Glycoproteomics for prostate cancer detection changes in serum PSA glycosylation patterns. J Proteome.

[23] Thuy TT, Thorsen G (2013). Glycosylation profiling of therapeutic antibodies in serum samples using a microfluidic CD platform and MALDI-MS. J Am Soc Mass Spectrom.

[24] Thuy TT, Inganas M, Thorsen G (2011). High-throughput profiling of N-linked oligosaccharides in therapeutic antibodies using a microfluidic CD platform and MALDI-MS. Anal Bioanal Chem.

[25] Lofblom J, Feldwisch J, Tolmachev V, Carlsson J, Stahl S, Frejd FY (2010). Affibody molecules: engineered proteins for therapeutic, diagnostic and biotechnological applications. FEBS Lett.

[26] Thuy TT, Tengstrand E, Aberg M, Thorsén G (2012). Discrimination between glycosylation patterns of therapeutic antibodies using a microfluidic platform, MALDI-MS and multivariate statistics. J Pharm Biomed Anal.

[27] Thuy TT, Inganas M, Ekstrand G, Thorsen G (2010). Parallel sample preparation of proteins, from crude samples to crystals ready for MALDI-MS, in an integrated microfluidic system. J Chromatogr B Anal Technol Biomed Life Sci.

[28] Bynum MA, Yin H, Felts K, Lee YM, Monell C, Killeen K (2009). Characterization of IgG N-glycans employing a microfluidic chip that integrates glycan cleavage, sample purification, LC separation, and MS detection. Anal Chem.

[29] Doherty M, Bones J, McLoughlin N, Telford JE, Harmon B, DeFelippis MR (2013). An automated robotic platform for rapid profiling oligosaccharide analysis of monoclonal antibodies directly from cell culture. Anal Biochem.

[30] Landberg E, Astrom E, Kagedal B, Pahlsson P (2012). Disialo-trisialo bridging of transferrin is due to increased branching and fucosylation of the carbohydrate moiety. Clin Chim Acta Int J Clin Chem.

[31] Beck A, Wagner-Rousset E, Ayoub D, Van Dorsselaer A, Sanglier-Cianferani S (2013). Characterization of therapeutic antibodies and related products. Anal Chem.

[32] Lin Z, Simeone DM, Anderson MA, Brand RE, Xie X, Shedden KA (2011). Mass spectrometric assay for analysis of haptoglobin fucosylation in pancreatic cancer. J Proteome Res.

[33] Shariatgorji M, Nilsson A, Goodwin RJ, Kallback P, Schintu N, Zhang X (2014). Direct targeted quantitative molecular imaging of neurotransmitters in brain tissue sections. Neuron.

[34] Gusev AI, Wilkinson WR, Proctor A, Hercules DM (1995). Improvement of signal reproducibility and matrix/comatrix effects in MALDI analysis. Anal Chem.

[35] Sroka-Bartnicka A, Olejniczak S, Sochacki M, Biela T, Potrzebowski MJ (2009). Solid-state NMR spectroscopy as a tool supporting optimization of MALDI-TOF MS analysis of polylactides. J Am Soc Mass Spectrom.

[36] Sroka-Bartnicka A, Cesielski W, Libiszowski J, Duda A, Sochacki M, Potrzebowski MJ (2010). Complementarity of solvent-free MALDI TOF and solid-state NMR spectroscopy in spectral analysis of polylactides. Anal Chem.

[37] Fagerer SR, Schmid T, Ibanez AJ, Pabst M, Steinhoff R, Jefimovs K (2013). Analysis of single algal cells by combining mass spectrometry with Raman and fluorescence mapping. Analyst.

[38] Nishikaze T, Okumura H, Jinmei H, Amano J (2012). Correlation between sweet spots of glycopeptides and polymorphism of the matrix crystal in MALDI samples. Mass Spectrom (Tokyo).

[39] Dai Y, Whittal RM, Li L (1996). Confocal fluorescence microscopic imaging for investigating the analyte distribution in MALDI matrices. Anal Chem.

[40] Bouschen W, Spengler B (2007). Artifacts of MALDI sample preparation investigated by high-resolution scanning microprobe matrix-assisted laser desorption/ionization (SMALDI) imaging mass spectrometry. Int J Mass Spectrom.

[41] Nishikaze T, Okumura H, Jinmei H, Amano J (2013). Advantages of pyrene derivatization to site-specific glycosylation analysis on MALDI mass spectrometry. Int J Mass Spectrom.

[42] Knochenmuss R, Zenobi R (2003). MALDI ionization: the role of in-plume processes. Chem Rev.

[43] Wold S, Esbensen K, Geladi P (1987). Principal component analysis. Chemom Intell Lab Syst.

[44] Wold S, Sjöström M. SIMCA: a method for analyzing chemical data in terms of similarity and analogy. In: Kowalski B, editor. Chemometrics, theory and application. Amer Chem Soc Symp.; 1977. p. 243–82.

[45] Sillanaukee P, Massot N, Jousilahti P, Vartiainen E, Poikolainen K, Olsson U (2000). Enhanced clinical utility of gamma-CDT in a general population. Alcohol Clin Exp Res.

[46] Sillanaukee P, Olsson U (2001). Improved diagnostic classification of alcohol abusers by combining carbohydrate-deficient transferrin and γ-glutamyltransferase. Clin Chem.

[47] Salaspuro M (1999). Carbohydrate-deficient transferrin as compared to other markers of alcoholism: a systematic review. Alcohol.

